# Supplementary figures and images for: Poly(ADP-ribose) polymerase 1 searches DNA via a ‘monkey bar’ mechanism
Source: eLife. 2018 Aug 8;7:e37818. doi: 10.7554/eLife.37818 (PMC6135609; doi:10.7554/eLife.37818)

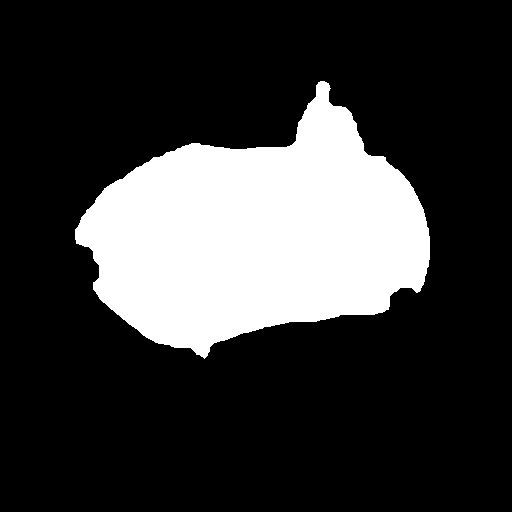

Supplement: Source code 1. [file elife-37818-code1.mltbx › metadata/primaryScreenShot.png]

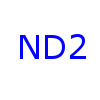

Supplement: Source code 2. [file elife-37818-code2.mltbx › metadata/primaryScreenShot.png]
